# Alteration of static and dynamic intrinsic brain activity induced by short-term spinal cord stimulation in postherpetic neuralgia patients

**DOI:** 10.3389/fnins.2023.1254514

**Published:** 2023-10-09

**Authors:** Chunxiao Bu, Huan Ren, Qingqing Lv, Huilian Bu, Xinyu Gao, Ruiping Zheng, Huiyu Huang, Weijian Wang, Yarui Wei, Jingliang Cheng, Yong Zhang

**Affiliations:** ^1^Department of Magnetic Resonance Imaging, The First Affiliated Hospital of Zhengzhou University, Zhengzhou, China; ^2^Department of Pain Medicine, Peking University Shenzhen Hospital, Shenzhen, China; ^3^Department of Radiology, The Third Affiliated Hospital of Zhengzhou University, Zhengzhou, China; ^4^Department of Pain Medicine, The First Affiliated Hospital of Zhengzhou University, Zhengzhou, China

**Keywords:** fMRI, amplitude of low-frequency fluctuation, dynamic amplitude of low-frequency fluctuation, spinal cord stimulation, postherpetic neuralgia

## Abstract

**Introduction:**

Short-term spinal cord stimulation (stSCS) is an effective treatment for postherpetic neuralgia (PHN). However, how exactly stSCS affects time-dynamic intrinsic brain activity in PHN patients is not clear. The purpose of this study was to examine the static and dynamic variability of neural activity in PHN patients after stSCS.

**Methods:**

In this study, 10 patients with PHN underwent resting-state functional magnetic resonance imaging (rs-fMRI) at baseline and after SCS. The amplitude of low-frequency fluctuations (ALFF) and dynamic ALFF (dALFF) were used to investigate the static and dynamic variability of neural activity in PHN patients after stSCS. We additionally examined the associations between clinical parameters and functional changes in the brain.

**Results:**

There was a significant increase in dALFF in the left precuneus and right superior parietal gyrus, and a decrease in dALFF in the left inferior temporal gyrus, right gyrus rectus, left superior temporal gyrus, right orbitofrontal cortex, and left orbitofrontal cortex. There was significantly increased ALFF in the right inferior temporal gyrus, and decreased ALFF in the right lingual gyrus, left superior parietal gyrus, right superior parietal gyrus, and left precuneus. Furthermore, Pittsburgh sleep quality index scores were positively associated with dALFF changes in the left superior temporal gyrus and left orbitofrontal cortex. Hospital anxiety and depression scale scores and continuous pain scores exhibited significant negative correlation with dALFF changes in the right superior parietal gyrus.

**Conclusion:**

This study indicated that stSCS is able to cause dALFF changes in PHN patients, thus stSCS might alter brain functions to relieve pain, sleep, and mood symptoms. The findings provide new insights into the mechanisms of stSCS efficacy in the treatment of patients with PHN.

## 1. Introduction

Herpes zoster (HZ) is caused by the reactivation of varicella-zoster virus and typically presents as a painful blister-like rash (Altena et al., 2010). The annual incidence of HZ is about 3.4/1000, rising sharply from the age of 50 to about 11/1000 by the ninth decade of life (Argaman et al., 2020). Postherpetic neuralgia (PHN) is the most common chronic complication of HZ, with pain appearing or persisting for around 1 to 3 months after the onset of the rash (Briggs et al., 2020). After 50 years of age, about 20% of patients with HZ will develop PHN (Brisson et al., 2001). PHN remains an important public health problem that leads to suffering and a reduced quality of life, and raises the cost of individual and societal health care (Buckner et al., 2008).

Because of the complexity of its pathogenesis, traditional pharmacological therapy is not always effective in relieving pain and may lead to a variety of drug-related complications (Borsook, 2012). stSCS whose electrodes are placed percutaneously to the spinal epidural for 2 weeks, and stSCS is known to be a clinically effective treatment option for these PHN patients. Since the introduction of spinal cord stimulation (SCS) as a therapeutic option for PHN, the mechanism of pain relief by SCS has been investigated. Multiple spinal segmental and supraspinal structures may play roles in the pain-alleviating effects of SCS (Drolet et al., 2010; Borsook, 2012; Deogaonkar et al., 2016). However, the potential functional alterations occurring in the brain as a result of SCS are less clear. Functional magnetic resonance imaging (fMRI) is a powerful noninvasive tool for understanding and mapping brain areas associated with pain perception and modulation (Deogaonkar et al., 2016). Moreover, the amplitude of low-frequency fluctuations (ALFF) can effectively reflect spontaneous brain activity and thus has been examined in PHN-related research. For example, Gu et al. (2019) observed a prominent decrease in ALFF in the right prefrontal cortex and increased ALFF in the bilateral brainstem and cerebellum anterior lobe in PHN patients. However, when HZ developed into PHN, neural activity was significantly increased in large areas of the cerebellum and frontal lobe but significantly decreased in the occipital lobe and limbic system (Cao et al., 2018). Only a few studies have used neuroimaging methods to explore the changes in brain activity after pain relief (Cao et al., 2018).

Based on resting-state functional MRI (rs-fMRI) evidence, ALFF was shown to reflect the average intrinsic activity of the brain over the entire scan. Although a previous study found abnormal ALFF in PHN patients, the neural activity of the brain is highly dynamic (Wen et al., 2021). ALFF only is not sufficient to describe the dynamic variability of spontaneous brain activity. Most previous studies have focused on static changes to neural activity in PHN patients (Cao et al., 2018; Gu et al., 2019; Zhang et al., 2020), and it is not sufficient to focus only on static changes in brain connectivity (Chang and Glover, 2010). Dynamic ALFF (dALFF) captures the temporal variability of the spontaneous neural activity of the brain with a sliding window that reflects changes in the information over the temporal dimension (Liao et al., 2019). Currently, changes in dynamic spontaneous brain activity occurring after SCS therapy in patients with PHN have not been evaluated. We hypothesized that, after stSCS, patients with PHN may present static and dynamic brain activity changes in some brain regions. We thus examined whether there are associations between clinical data and functional brain mapping changes in PHN patients after stSCS.

## 2. Materials and methods

### 2.1. Participants

We recruited 19 right-handed PHN patients from our pain department between January and October 2021 whose pain was not relieved by conventional medication alone and, therefore, were treated with stSCS at the First Affiliated Hospital of Zhengzhou University. All participants signed a written informed consent prior to participation in this study. Nine patients withdrew their consent or had poor quality MRI images; therefore, the final valid data from our study were obtained from 10 patients.

This study was approved by the Medical Ethics Committee of the First Affiliated Hospital of Zhengzhou University (Reference: 2020-KY-0299-001). The trial registration number and web address are ChiCTR2000040239.^1^

### 2.2. Research protocol

In this prospective cohort study, patients were enrolled pre-stSCS implantation and followed for 14 days after receiving stSCS. During admission, clinical scales were assessed prior to MRI, which took 30 min, and patients were asked to complete questions relating to the pain numerical rating scale (NRS), the short-form McGill pain questionnaire version-2 (SF-MPQ-2), the Pittsburgh sleep quality index (PSQI), and the hospital anxiety and depression scale (HADS). The SF-MPQ-2 involves four subscale scores (continuous pain, intermittent pain, predominantly neuropathic pain, and affective descriptors). Electrodes were removed 14 days after SCS implantation, and all patients underwent neuroimaging with an fMRI-protocol before (baseline) and 14 days after SCS implantation.

All patients received SCS at the cervicothoracic level for PHN. For the detailed methodology of SCS used in the study, please refer to the previous study by Fan et al. (2022). Neurostimulation test electrodes (Model 3,873, US) were implanted under dynamic monitoring with DSA imaging, then connected to an extension cable (Model 355,531, Medtronic, US) and external neurostimulator (Model 37,022, Medtronic, US). The physician adjusted the parameters to determine the effective contact, and the patient was free to change the amplitude in 0.1 mV increments to provide adequate pain relief. The stimulation parameters were as follows: voltage 0–10.5 V, pulse width 210–480 μs, frequency 30–60 Hz. Spinal electrical stimulation was applied for 14 consecutive days.

### 2.3. MRI image acquisition

All MRI images were acquired with a 3-T Siemens MR scanner (Magnetom Prisma, Siemens, Germany) at the magnetic resonance department of the First Affiliated Hospital of Zhengzhou University. During the scans, all subjects were asked to close their eyes and try not to think about anything else. Foam pads were applied to control the subjects’ head movements, and earmuffs were fixed on both ears to reduce the noise made by the scanner. At the end of the scan, patients were asked whether they had fallen asleep during the scan. All patients reported that they had been awake at all times. The rs-fMRI was acquired using an echo-planar imaging sequence, and the scanning parameters were as follows: repetition time (TR) = 1,000 ms, echo time (TE) = 30 ms, flip angle = 70°, slice number = 52, field of view = 220 × 220 mm^2^, matrix = 110 × 110, slice thickness = 2.2 mm, 400 volumes, scan time = 412 s.

### 2.4. MRI data processing

The Data Processing Assistant for rs-fMRI Analysis Toolbox (DPARSF, http://rest.Restfmri.Net/forum/DPARSF; Chao-Gan and Yu-Feng, 2010; Song et al., 2011) and SPM8 software (Wellcome Department, University College of London, UK) based on MATLAB R2012a (MathWorks, USA) were used to preprocess the rs-fMRI data. The main steps were as follows: (1) removal of initial 10 volumes to ensure signal stability; (2) slice timing and realignment; (3) spatial normalization to the standard Montreal Neurological Institute (MNI) space and resampling with a resolution of 3 × 3 × 3 mm^3^; (4) detrending: multiple linear regression analysis was used to regress several spurious variances, including global mean signals, white matter signals, cerebrospinal fluid signals, and Friston-24 head motion parameters; (5) scrubbing of the image volumes with frame-wise displacement, (FD) > 0.5 mm, using spline interpolation to reduce the influence of head motion; (6) spatial smoothing of functional images with a full-width Gaussian kernel at half-maximum of 6 mm; (7) removal of high-frequency physiological noise and frequency drift lower than 0.01 Hz using band-pass filtering (0.01–0.08 Hz; Greicius et al., 2003).

### 2.5. Imaging analysis

The dALFF analysis data were analyzed using the Dynamic Brain Connectome toolbox (Liao et al., 2014; v2.0, http://restfmri.net/forum/DynamicBC). A sliding window approach was applied to characterize the temporal dynamic modes. Window length is an important parameter in the calculation of resting state dynamics, and the range of window lengths should be small enough to detect potential transients but large enough to analyze small fluctuations of interest in ALFF (Sakoglu et al., 2010; Zheng et al., 2021). A previous study (Leonardi and Van De Ville, 2015) showed that, to avoid introducing false fluctuations, a frequency interval of [0–1/w] Hz should be targeted, and the minimum window length should be 1/fmin. Therefore, we chose a window size of 30 TRs (30s) and a window overlap of 80% to calculate the dALFF of each subject. In addition, we calculated the results with other window sizes and overlaps and included them in the validation analyses. To explore the correlations between abnormal intrinsic timescales and clinical outcomes, we extracted the average intrinsic timescale values of all voxels within each cluster from the corrected statistical plots.

### 2.6. Validation analysis

To validate our main results, we tested the differences between the results and those obtained using other window lengths (50 TRs, 0.8 overlap, 80 TRs, 0.8 overlap) and different overlap rates.

### 2.7. Statistical analysis

To explore changes in the dynamic variability of dALFF, paired *t*-tests were performed on dALFF data between baseline and after stSCS with SPM8 in a whole-brain voxel-wise manner. Gaussian random field (GRF) was used to correct all results. The significance levels of the voxel and cluster were set at *p* < 0.005 and *p* < 0.05, respectively, and the minimum cluster size was 30 voxels. When significant differences in dALFF were observed in any regions of the brain, we extracted the mean dALFF values of the region of interest for each dALFF using the toolkit rs-fMRI analysis (http://www.restfmri.net/forum/REST; Song et al., 2011). The relationships between the mean values (dALFF variability) and clinical variables (NRS, SF-MQP-2, PSQI, HADS score) were then further assessed using the non-parametric Spearman correlation test. The threshold for all correlation analyses was *p* < 0.05/108 (Bonferroni corrected), which was statistically significant.

## 3. Results

### 3.1. Patient characteristics

Ten patients with PHN (five females and five males) with a median age of 70 years were included in our study. The demographics and clinical features of the patients are summarized in Table 1.

**Table 1:** Individual characteristics of patients included in this study (n = 10).

Patients ID	Sex(F/M)	Age (years)	Pain side	Pain duration (month)	Duration of SCS (days)
1	F	62	Left	6	14
2	F	79	Left	3	14
3	F	70	Right	2	14
4	F	68	Left	4	14
5	F	87	Right	1.5	14
6	M	39	Right	6	14
7	M	60	Right	7	14
8	M	70	Left	1	14
9	M	77	Left	1	14
10	M	83	Left	1	14

M: male, F: female, SCS: spinal cord stimulation.

### 3.2. Clinical results

**Figure 1:**
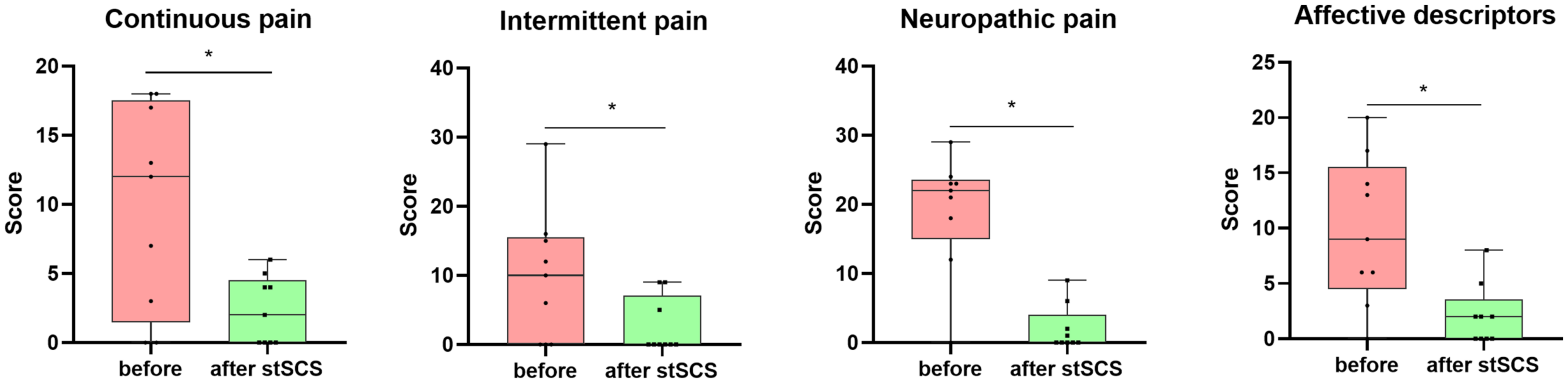
Boxplots showing the clinical SF-MPQ-2 scores of all patients at baseline (in pink) and after stSCS (in green). The four boxplots represent the four subscale scores of the SF-MPQ-2 (continuous pain, intermittent pain, predominantly neuropathic pain, and affective descriptors). SCS, spinal cord stimulation.

**Figure 2:**
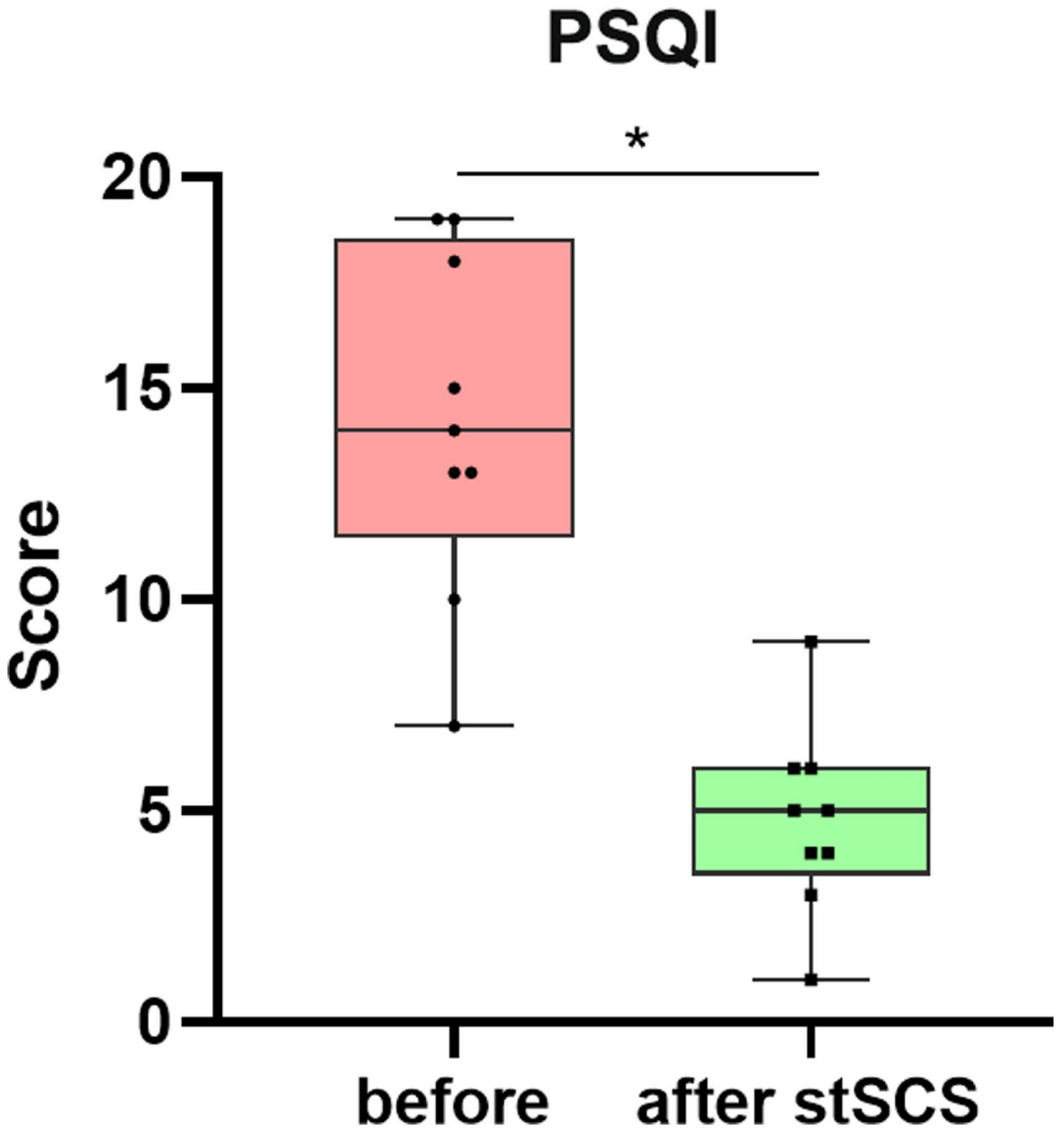
Boxplot showing the clinical PSQI scores of patients at baseline (in pink) and after stSCS (in green). PSQI, Pittsburgh sleep quality index; SCS, spinal cord stimulation.

**Figure 3:**
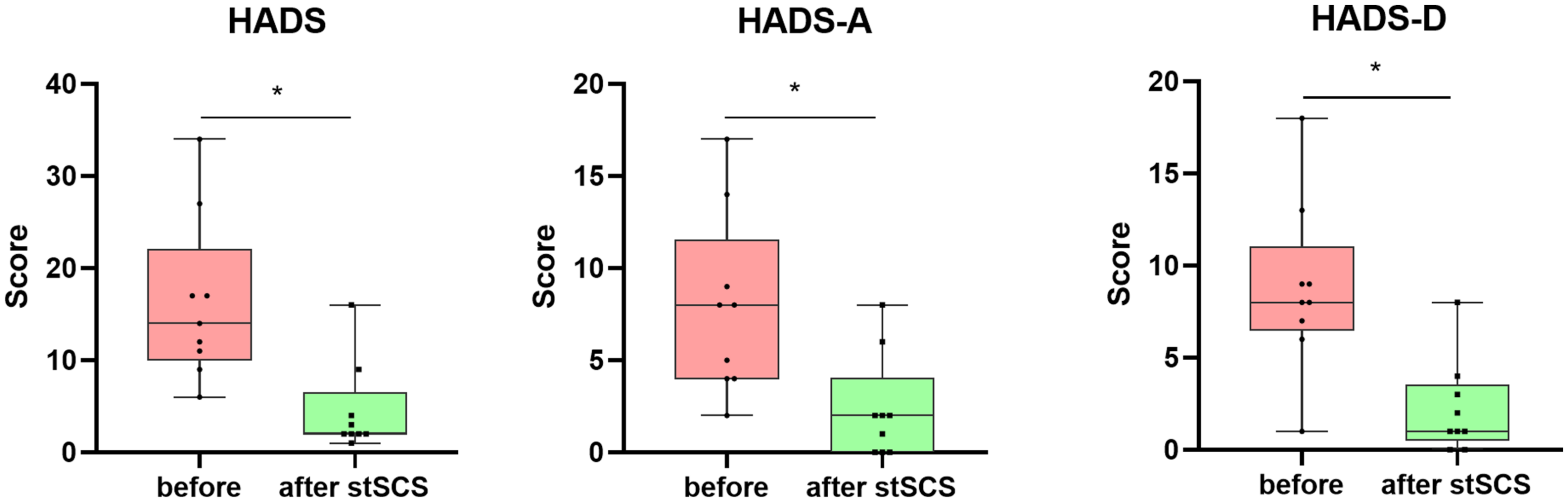
Boxplots showing the clinical HADS scores of patients at baseline (in pink) and after stSCS (in green). The second and third boxplots represent the two subscale scores for the HADS (HADS-A and HADS-D, respectively). HADS, hospital anxiety and depression scale; HADS-A: hospital anxiety and depression scale-anxiety: HADS-D: hospital anxiety and depression scale-depression; SCS: spinal cord stimulation.

**Figure 4:**
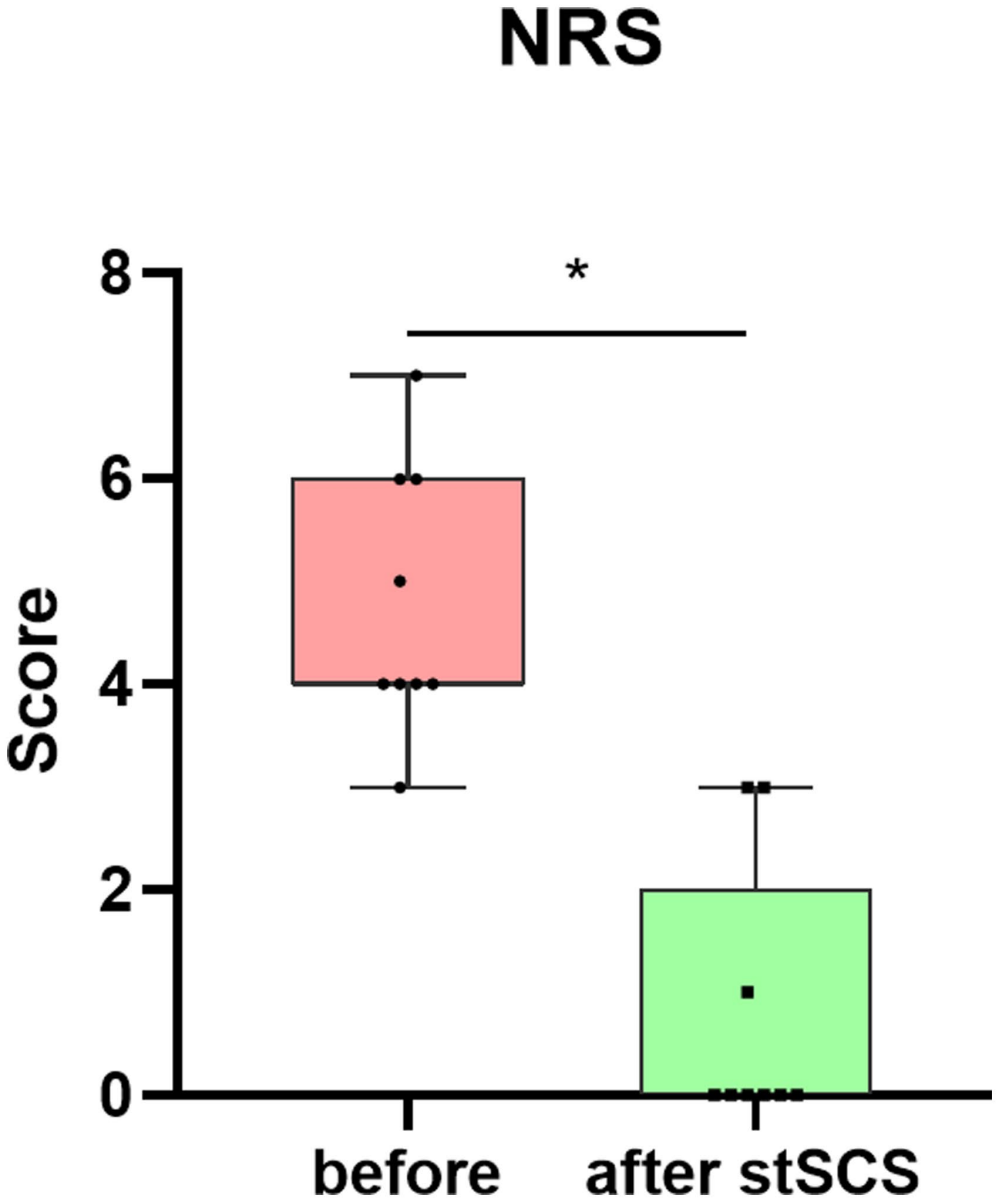
Boxplot showing the clinical NRS scores of patients at baseline (in pink) and after stSCS (in green). NRS, numerical rating scale; SCS, spinal cord stimulation.

There were significantly reduced total scores for SF-MPQ-2(Z = −2.694, *p* = 0.007), PSQI (Z = −2.675, *p* = 0.007), HADS (Z = −2.668, *p* = 0.008), and NRS (Z = −2.694, *p* = 0.007) after stSCS compared with the baseline (Figures 1–4). When examining continuous pain, intermittent pain, neuropathic pain, and affective descriptors of SF-MPQ-2, we found that the scores significantly decreased after stSCS (Z = −2.371, *p* = 0.018; Z = −2.207, *p* = 0.027; Z = −2.527, *p* = 0.012; and Z = −2.524, *p* = 0.012, respectively), as displayed in Figure 1. Concerning the anxiety and depression aspects of HADS, we found a significant decrease in HADS-A and HADS-D scores after stSCS (Z = −2.677, *p* = 0.007; and Z = −2.673, *p* = 0.008, respectively), as presented in Figure 3.

### 3.3. fMRI results

#### 3.3.1. dALFF and ALFF results

**Figure 5:**
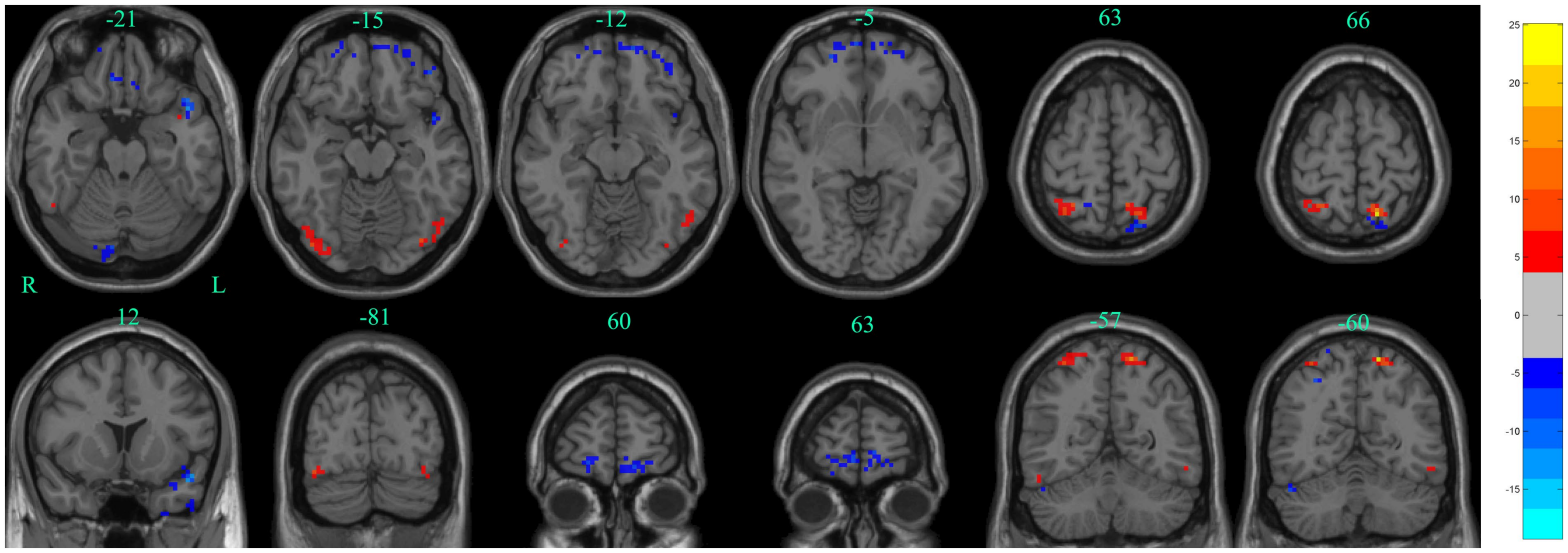
Significant differences in dALFF between baseline and after stSCS in axial and coronal slices (30 TRs, 80% overlap). Warm colors indicate higher dALFF values, while cooler colors indicate lower dALFF values at baseline and after stSCS. The statistical significance level was set at *p*_voxel_ < 0.005, *p*_cluster_ < 0.05 (GRF corrected).

The main results of the study were based on dALFF analysis using 30 TRs and 80% overlap and are displayed in Table 2 and Figure 5. Compared to baseline, dALFF was significantly increased in the left precuneus and right superior parietal gyrus, but decreased in the left inferior temporal gyrus, right gyrus rectus, left superior temporal gyrus, right orbitofrontal cortex, and left orbitofrontal cortex (GRF corrected *p*_voxel_ < 0.005, *p*_cluster_ < 0.05).

**Table 2:** Different dALFF values between baseline and after stSCS.

Regions	MNI coordinate			Peak *T* value	Voxels
	x	y	z		
dALFF increase					
Left precuneus	−15	−60	66	25.1361	82
Right superior parietal gyrus	33	−57	63	9.741	42
dALFF decreased					
Left inferior temporal gyrus	−48	0	−42	−6.3794	37
Right gyrus rectus	9	33	−21	−8.1885	65
Left superior temporal gyrus	−45	12	−21	−14.0498	40
Right orbitofrontal cortex	6	63	−3	−8.7597	42
Left orbitofrontal cortex	−12	60	−12	−6.99	66

dALFF, dynamic amplitude of low-frequency fluctuation; MNI, Montreal Neurological Institute.

**Figure 6:**
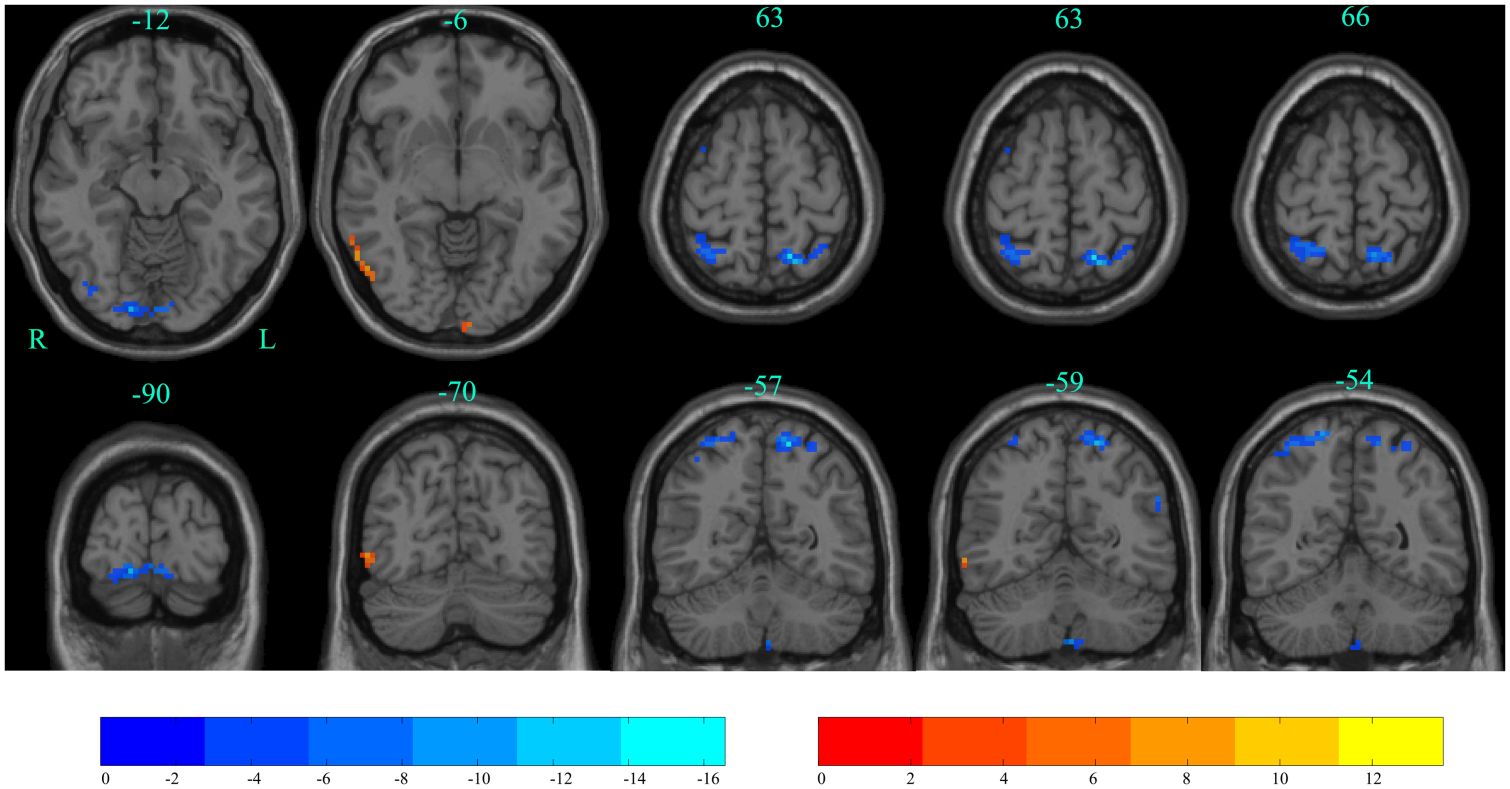
Significant differences in ALFF between baseline and after stSCS in axial and coronal slices. Warm colors indicate higher ALFF values, while cooler colors indicate lower ALFF values at baseline and after stSCS. The statistical significance level was set at *p*_voxel_ < 0.005, *p*_cluster_ < 0.05 (GRF corrected).

The differences between ALFF at baseline and after stSCS of static ALFF patterns are shown in Table 3 and Figure 6. There was significantly elevated ALFF in the right inferior temporal gyrus; and a decrease in ALFF in the right lingual gyrus, left superior parietal gyrus, right superior parietal gyrus, and left precuneus (GRF corrected *p*_voxel_ < 0.005, *p*_cluster_ < 0.05).

**Table 3:** Different ALFF values between baseline and after stSCS.

Regions	MNI coordinate			Peak *T* value	Voxels
	x	y	z		
ALFF increase					
Right inferior temporal gyrus	51	−72	−12	8.6165	32
ALFF decreased					
Right lingual gyrus	15	−90	−12	−11.1194	70
Left superior parietal gyrus	−27	−72	57	−7.5537	45
Right superior parietal gyrus	18	−54	69	−10.1811	53
Left precuneus	−15	−57	63	−16.5591	45

ALFF, amplitude of low-frequency fluctuation; MNI, Montreal Neurological Institute.

#### 3.3.2. Correlation analyses

The PSQI score was positively associated with the dALFF values in the right gyrus rectus, the left superior temporal gyrus, and the left orbitofrontal cortex (*r* = 0.746, *p* = 0.021; *r* = 0.729, *p* = 0.026; *r* = 0.678, *p* = 0.045, respectively). The HADS-D score and continuous pain score were significantly negatively correlated with dALFF changes in the right superior parietal gyrus (r = −0.700, *p* = 0.036; r = −0.689, *p* = 0.040, respectively). The HADS-A score was positively associated with ALFF values in the right lingual gyrus (r = 0.820, *p* = 0.007). Neuropathic pain and affective descriptors were significantly positively correlated with ALFF changes in the right superior parietal gyrus and the left precuneus (r = 0.866, *p* = 0.003; r = 0.845, *p* = 0.004; r = 0.765, *p* = 0.016; r = 0.820, *p* = 0.007; respectively). However, this difference in significance level was cancelled out after Bonferroni calibration (*p* < 0.05/108 = 0.000463).

#### 3.3.3. Validation analyses

In our study, the different sliding window lengths and different overlap rates were applied to validate our results. The results of different overlap (30 TRs, 0.6 overlap) and the other two window lengths (50 TRs, 0.8 overlap; 80 TRs, 0.8 overlap) are presented in Supplementary Tables 1–3. Supplementary Figures 1–3. These results were generally consistent with our main results.

## 4. Discussion

Researchers have found that dALFF can provide evidence for the dynamic variability of spontaneous brain activity in the brain, and it has provided us with a new way to explore fluctuations in spontaneous brain activity in PHN patients after stSCS. In our research, we studied changes in the dALFF and ALFF values of brain regions associated with pain relief after stSCS in PHN patients. The dALFF and ALFF analyses verified that the improvement of symptoms was, to some extent, associated with altered regional brain functions. Our study also assessed the relationship between changes in brain function and clinical variables, and our findings suggest that stSCS alters the statics and dynamics of local neural activity to relieve pain, sleep, and mood disorders.

In the present study, stSCS can rapidly and effectively relieve pain in PHN patients, which is consistent with previous results (Dong et al., 2017; Feng and Ye, 2021; Sheng et al., 2022). Moreover, stSCS can effectively improve sleep quality and emotion in patients (Feng and Ye, 2021; Liu et al., 2021). In addition, we found that, after stSCS treatment, PHN patients had functional alterations in several brain regions that may be associated with pain relief.

In this study, we observed changes in several brain functions associated with sleep quality after stSCS treatment of PHN patients. The main clinical features of patients with neuropathic pain are the symptoms of mood disorders, such as anxiety, depression, and insomnia (Inoue et al., 2017). Previous studies have suggested that neuropathic pain and affective disorders may share a common pathogenesis (Aloisi et al., 2016). PHN patients in our study also experienced varying degrees of depression and poor sleep quality, but experienced a mood boost and improved sleep after stSCS. Our study found that dALFF decreased after treatment in the left orbitofrontal cortex, right orbitofrontal cortex, and right gyrus rectus. In addition, we also found that changes in dALFF in the left orbitofrontal cortex and right gyrus rectus were positively correlated with PSQI. The orbitofrontal cortex (OFC) is mainly involved in sensory integration and monitoring the responses of internal organs and the internal state of the body, as well as evaluating sensory responses and regulating autonomic responses (Kringelbach, 2005; Vandenberghe et al., 2007). In addition, the orbitofrontal cortex is an important region known to be involved in pain processing at multiple levels, influencing bidding behavior and decision-making, and mediating pain inhibition (Mouraux et al., 2011; Winston et al., 2014). A study showed back and leg pain patients to have increased ALFF in their bilateral OFC (Zhou et al., 2018). Therefore, we concluded that decreased intrinsic activity in the OFC may reduce pain in PHN patients. The OFC also plays an important role in sleep. Park et al. (2021) reported that reduced cerebral blood flow in the orbitofrontal and insular cortices was associated with poor sleep quality. We reasoned that one of the mechanisms behind the improvement in sleep may be related to changes in the OFC.

In this study, we identified the superior parietal gyrus as being related to pain intensity and HADS-D after the treatment of PHN with stSCS. The parietal gyrus is involved in the processing of emotional, sensory, and cognitive functions of the brain (Lai, 2019). Some studies have found that the onset of anxiety is associated with reduced cerebral blood flow in the parietal gyrus (Fredrikson et al., 1997). Dai et al. (2020) demonstrated that PHN patients had increased fALFF in the left cerebellum posterior gyrus, left orbital gyrus, and right superior parietal gyrus. Yue and Du (2020) found increased cerebral blood flow (CBF) in the bilateral superior parietal gyri in patients with chronic neck pain, which might be a compensatory manifestation. In our study, we found decreased ALFF values in the bilateral superior parietal gyri after stSCS, and there was a positive correlation between ALFF values, neuropathic pain, and affective descriptors. The superior parietal gyrus is believed to be associated with visuospatial attention (Wu et al., 2016). Furthermore, it has been shown that the stronger the spontaneous activity in the superior parietal gyrus, the higher the level of pain experienced by the patient (Li et al., 2021).

We discovered that the precuneus is associated with the modulation of pain intensity. We noticed a decrease in ALFF values in the left precuneus after stSCS compared to baseline. Significant correlations were also found between changes in ALFF in the left precuneus and changes in neuropathic pain and affective descriptors. The precuneus is responsible for collecting information about somatosensory sensations and is therefore primarily responsible for identifying pain sensations (Nagamachi et al., 2006). The precuneus is also involved in a range of highly integrated tasks, mainly including self-processing operations, visuospatial image processing, the processing of conscious information, pain perception, and the modulation of endogenous pain (Cavanna and Trimble, 2006; Greicius et al., 2009). De Groote et al. (2020) suggested that the precuneus could be used as a monitoring tool for the therapeutic effects of SCS. dALFF reflects the dynamic changes occurring in local spontaneous brain activity over time (Fu et al., 2018), and the greater the dALFF value, the more unstable the local spontaneous brain activity. In our study, we also observed an increase in dALFF values in the left precuneus, which may confirm the important role of the precuneus in pain reduction, and the precuneus showed fluctuations in regional spontaneous brain activity. The precuneus is the main center of the brain and one of the core regions of the default mode network (DMN). The DMN is the resting-state network of the brain and is mainly composed of the precuneus, the medial frontal gyrus, the posterior cingulate cortex, the posterior parietal cortex, and the lateral temporal cortex (Raichle et al., 2001). The DMN is responsible not only for emotional processing, self-introspection, and the extraction of awareness and scenario memory (Buckner et al., 2008) but also for associated pain inhibition (Argaman et al., 2020). In addition, DMN is a brain network and functional connection hub (Tomasi and Volkow, 2011). Patients with chronic pain show abnormalities in their resting DMN, suggesting that this chronic state affects areas beyond pain perception (Tagliazucchi et al., 2010; Otti et al., 2013; Zhang et al., 2014).

We observed decreased ALFF values in the right lingual gyrus after stSCS. The lingual gyrus is a part of the occipital lobe, and previous research has demonstrated that the lingual gyrus is also involved in activities related to visual memory processing and is strongly associated with the progression of major depressive disorder (Palejwala et al., 2021). A previous study also found that the right lingual gyrus is a sign of depression relief (Yang et al., 2018). Correlation analysis in our study showed that ALFF values in the right lingual gyrus were positively correlated with HADS and HADS-A scores. Therefore, we hypothesized that stSCS acts on the lingual gyrus to relieve depression and thus pain.

The main limitation of this study was the relatively small sampling size. This study was conducted over a long period of time, and it was difficult to collect cases due to the study design’s focus on treatment effects. In the future, a multi-center study will be conducted to collect more cases and obtain more reliable results. Furthermore, we investigated changes in brain function in patients who experienced pain relief after stSCS treatment; future studies will include a sham group to make the results more credible.

## 5. Conclusion

The findings of study suggest that stSCS may alter the local neural activity of ALFF and dALFF in key brain regions to alleviate pain, sleep, and emotional disorder in PHN patients. This study has provided further evidence of the neural mechanisms of stSCS in the treatment of PHN patients.

## Data availability statement

The original contributions presented in the study are included in the article/Supplementary material, further inquiries can be directed to the corresponding author.

## Ethics statement

The studies involving humans were approved by the Medical Ethics Committee of the First Affiliated Hospital of Zhengzhou University (Reference: 2020-KY-0299-001). The trial registration number and web address are ChiCTR2000040239 (http://www.chictr.org.cn/listbycreater.aspx). The studies were conducted in accordance with the local legislation and institutional requirements. The participants provided their written informed consent to participate in this study.

## Author contributions

CB: Writing – original draft, Writing – review & editing, Data curation, Formal analysis, Methodology, Software. HR: Data curation, Writing – review & editing, Formal analysis, Methodology. QL: Formal analysis, Investigation, Software, Writing – original draft. HB: Conceptualization, Data curation, Investigation, Writing – review & editing. XG: Conceptualization, Investigation, Software, Writing – review & editing. RZ: Conceptualization, Investigation, Software, Writing – review & editing. HH: Conceptualization, Investigation, Software, Writing – review & editing. WW: Formal analysis, Project administration, Validation, Writing – review & editing. YW: Supervision, Validation, Writing – review & editing. JC: Project administration, Validation, Writing – review & editing. YZ: Formal analysis, Project administration, Validation, Visualization, Writing – review & editing.

## Footnotes

^1^ http://www.chictr.org.cn/listbycreater.aspx
