# Supplementary material for: Alteration of static and dynamic intrinsic brain activity induced by short-term spinal cord stimulation in postherpetic neuralgia patients
Source: Front Neurosci. 2023 Oct 9;17:1254514. doi: 10.3389/fnins.2023.1254514 (PMC10590878; doi:10.3389/fnins.2023.1254514)
Supplement: Supplementary file 1 [file Data_Sheet_1.DOC]

***Supplementary Material***

**1 Supplementary Figures and Tables**

**1.1 Supplementary Tables**

**Supplementary Table 1.** Different dALFF values between baseline and after stSCS in 30 TRs and 60% overlap

| Regions | Peak MNI coordinate | | | Peak T value | Cluster size  (voxels) |
| --- | --- | --- | --- | --- | --- |
|  | x | y | Z |  |  |
| dALFF increase | | | | | |
| Left precuneus | −15 | −60 | 66 | 29.2382 | 57 |
| Right superior parietal gyrus | 27 | −54 | 66 | 10.6893 | 35 |
| dALFF decreased | | | | | |
| Left inferior temporal | −48 | 0 | −42 | −6.4237 | 33 |
| Right gyrus rectus | 9 | 33 | −21 | −7.6857 | 62 |
| Left superior temporal gyrus | −48 | 12 | −18 | −15.7284 | 39 |
| Right orbitofrontal cortex | 6 | 63 | −3 | −8.9551 | 37 |
| Left orbitofrontal cortex | −15 | 57 | −9 | −7.252 | 50 |

dALFF, dynamic amplitude of low-frequency fluctuation; MNI, Montreal Neurological Institute; L, left; R, right.

**Supplementary Table 2.** Different dALFF values between baseline and after stSCS in 50 TRs and 80% overlap

| Regions | Peak MNI coordinate | | | Peak T value | Cluster size  (voxels) |
| --- | --- | --- | --- | --- | --- |
|  | x | y | Z |  |  |
| dALFF increase | | | | | |
| Left precuneus | −15 | −57 | 66 | 25.1361 | 59 |
| dALFF decreased | | | | | |
| Left inferior temporal gyrus | −45 | 12 | −21 | −14.1562 | 37 |
| Right gyrus rectus | 9 | 33 | −21 | −7.4952 | 52 |
| Right postcentral gyrus | 63 | −15 | 15 | −10.6438 | 32 |
| Right angular gyrus | 30 | −60 | 51 | −8.0401 | 43 |

dALFF, dynamic amplitude of low-frequency fluctuation; MNI, Montreal Neurological Institute; L, left; R, right.

**Supplementary Table 3.**The different dALFF values between baseline and after stSCS in 80 TRs and 80% overlap

| Regions | Peak MNI coordinate | | | Peak T value | Cluster size  (voxels) |
| --- | --- | --- | --- | --- | --- |
|  | x | y | Z |  |  |
| dALFF increase | | | | | |
| Left superior parietal gyrus | −18 | −57 | 66 | 9.4336 | 40 |
| Right superior parietal gyrus | 33 | −60 | 63 | 13.1563 | 24 |
| dALFF decreased | | | | | |
| Left superior temporal gyrus | −45 | 12 | −21 | −13.8156 | 31 |
| Right gyrus rectus | 9 | 33 | −21 | −6.2228 | 31 |

dALFF, dynamic amplitude of low-frequency fluctuation; MNI, Montreal Neurological Institute; L, left; R, right.

**1.2 Supplementary Figures**


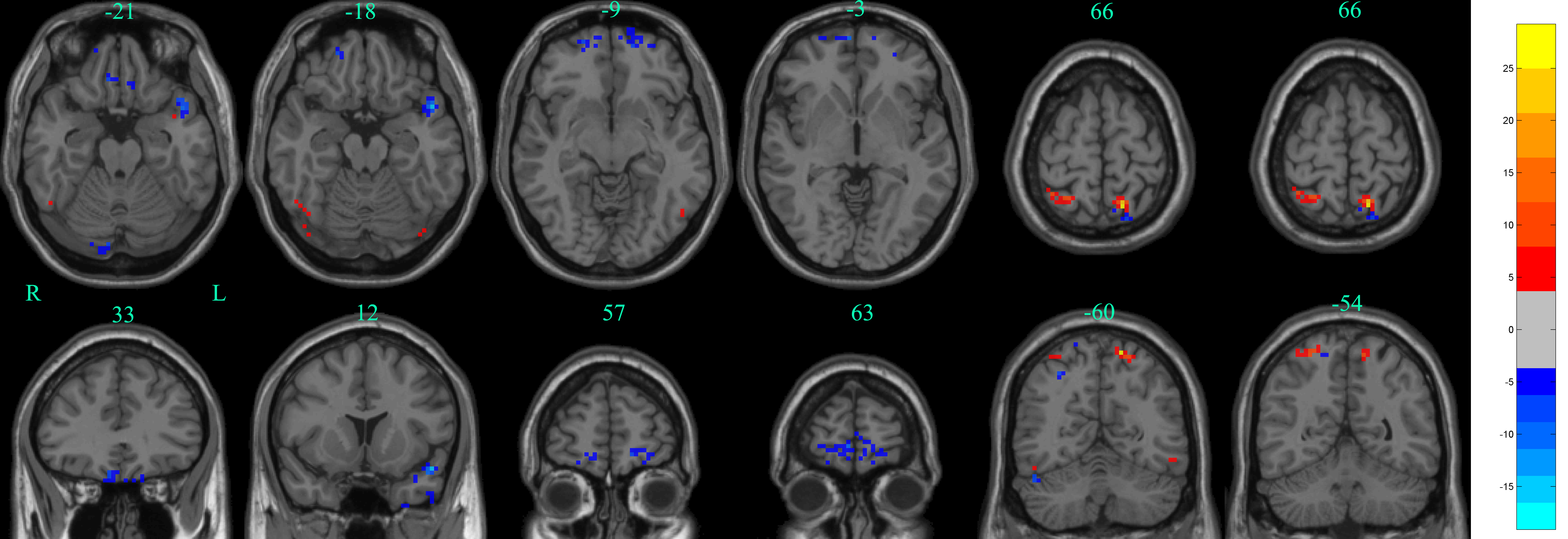


**Supplementary Figure 1. Significant differences in dALFF between baseline and after stSCS in axial and coronal slices (30 TRs, 60% overlap).** Warm colors indicate higher dALFF values while the cooler colors indicate lower dALFF values at baseline and after stSCS. The statistical significance level was set at *p*_voxel_ < 0.005, *p*_cluster_ < 0.05 (GRF corrected).


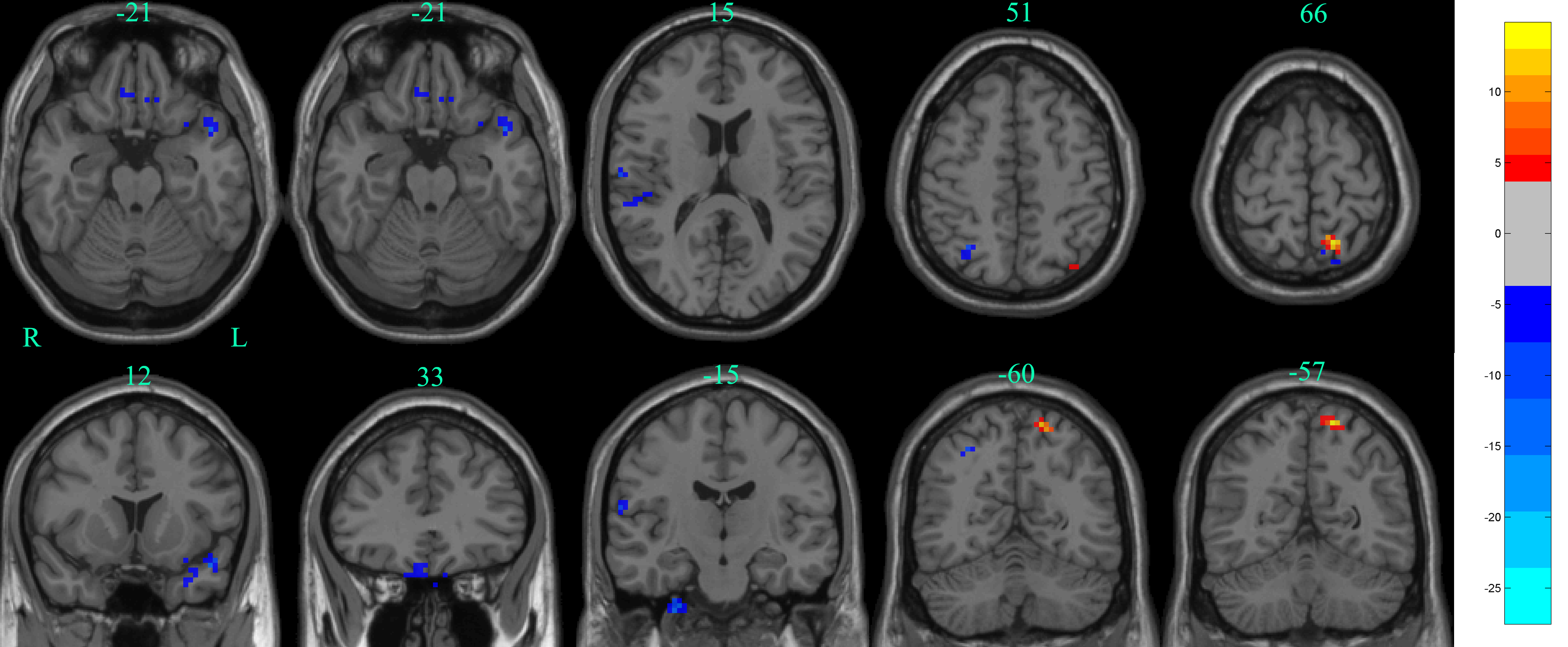


**Supplementary Figure 2. Significant differences in dALFF between baseline and after stSCS in axial and coronal slices (50 TRs, 80% overlap).** Warm colors indicate higher dALFF values while the cooler colors indicate lower dALFF values at baseline and after stSCS. The statistical significance level was set at *p*_voxel_ < 0.005, *p*_cluster_ < 0.05 (GRF corrected).


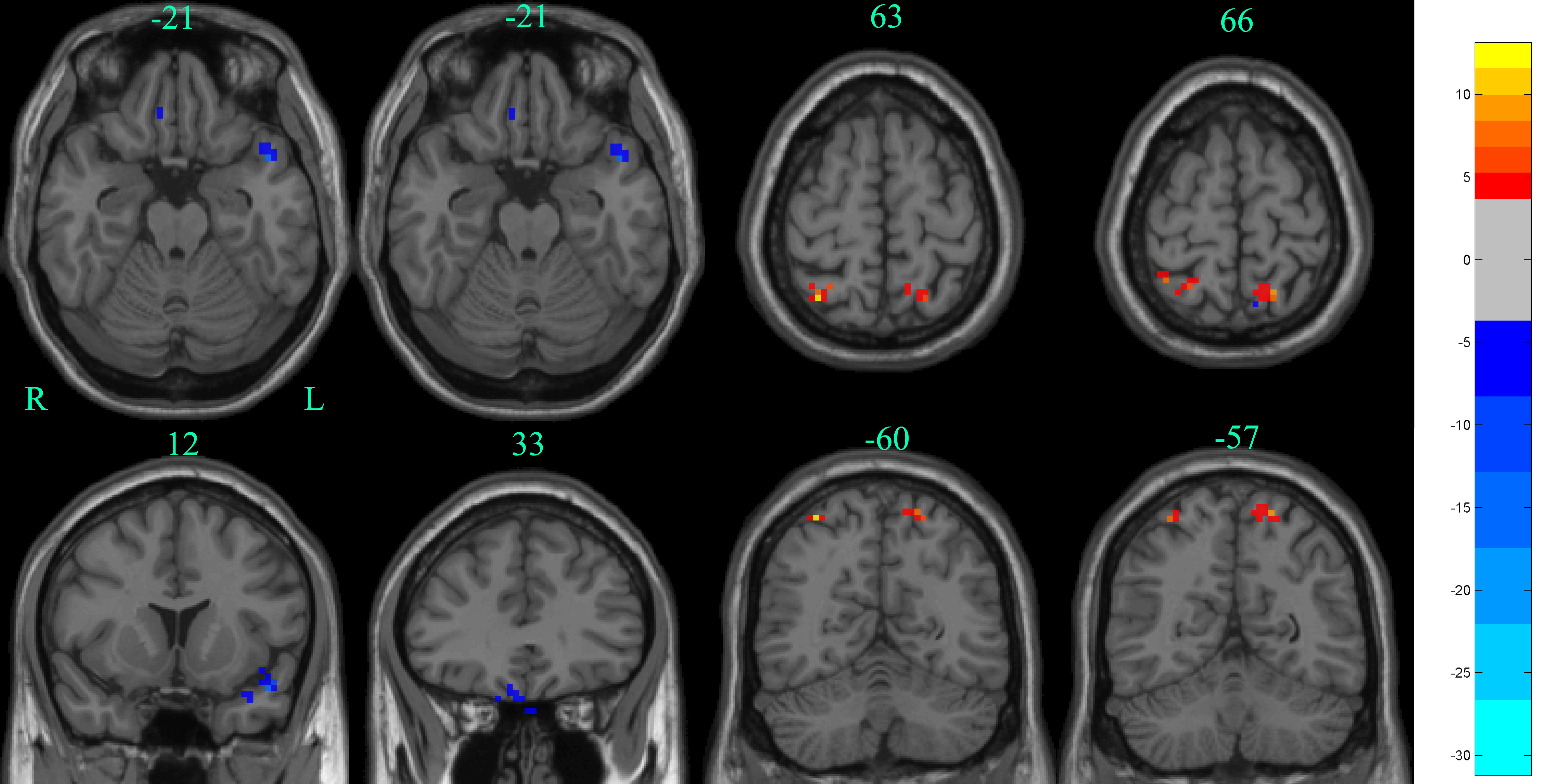


**Supplementary Figure 3. Significant differences in dALFF between baseline and after stSCS in axial and coronal slices (80 TRs, 80% overlap).** Warm colors indicate higher dALFF values while the cooler colors indicate lower dALFF values at baseline and after stSCS. The statistical significance level was set at *p*_voxel_ < 0.005, *p*_cluster_ < 0.05 (GRF corrected).
